# Anti-SAE autoantibody-positive Japanese patient with juvenile dermatomyositis complicated with interstitial lung disease - a case report

**DOI:** 10.1186/s12969-021-00532-2

**Published:** 2021-03-19

**Authors:** Takayuki Kishi, Yumi Tani, Naoko Okiyama, Kiyoshi Mizuochi, Yuki Ichimura, Masayoshi Harigai, Satoru Nagata, Takako Miyamae

**Affiliations:** 1 Department of Pediatrics, Tokyo Women’s Medical University, 8-1, Kawada-cho, Shinjuku-ku, Tokyo, 162-8666 Japan; 2 Department of Rheumatology, Tokyo Women’s Medical University, 8-1 Kawada-cho, Shinjuku-ku, Tokyo, 162-8666 Japan; 3 Department of Dermatology, Faculty of Medicine, University of Tsukuba, 1-1-1 Tennoudai, Tsukuba-shi, Ibaraki, 305-8575 Japan

**Keywords:** Juvenile dermatomyositis, Myositis-specific autoantibody, Interstitial lung disease, Anti-SAE autoantibody

## Abstract

**Background:**

Clinical phenotypes and outcomes in juvenile dermatomyositis (JDM) have been defined by various myositis-specific autoantibodies (MSAs). One of the recently described MSAs associated with DM is targeted against the small ubiquitin-like modifier 1 activating enzyme (SAE). We report an anti-SAE autoantibody-positive JDM patient complicated with interstitial lung disease (ILD).

**Case presentation:**

An 8-year-8-month-old Japanese girl presented with bilateral eyelid edema and facial erythema. At 8 years 4 months, she had dry cough and papules with erythema on the dorsal side of the interphalangeal joints of both hands. Her facial erythema gradually worsened and did not improve with topical steroids. At the first visit to our department at 8 years 8 months of age, she had a typical heliotrope rash and Gottron’s papules, with no fever or weight loss, and a chest computed tomography scan showed ground-glass opacity under visceral pleura. There was no clinical evidence of myositis, muscle weakness, myalgia, or muscle magnetic resonance imaging (MRI) findings. She had mild dry cough, without any signs of respiratory distress. Laboratory tests showed no elevated inflammatory markers. She had a normal serum creatine kinase level with a slightly elevated aldolase level, and serum anti-SAE autoantibody was detected by immunoprecipitation—western blotting. She was diagnosed with juvenile amyopathic DM complicated by ILD and received two courses of methylprednisolone pulse therapy followed by oral corticosteroid and cyclosporin A. We gradually reduced the corticosteroid dose as her skin rash improved after treatment initiation. There was no progression of muscle symptoms, dysphagia, or disease flare during a 24-month follow-up period.

**Conclusions:**

We report a patient with anti-SAE autoantibody-positive JDM complicated by interstitial pneumonia. This patient had no progression of muscle symptoms and dysphagia during a 24-month follow-up period, which differs from previous reports in adult patients with MSAs. There have been no previous reports of pediatric patients with SAE presenting with ILD. However, ILD seen in this case was not rapidly progressive and did not require cytotoxic agents. To prevent overtreatment, appropriate treatment choices are required considering the type of ILD.

**Supplementary Information:**

The online version contains supplementary material available at 10.1186/s12969-021-00532-2.

## Background

Clinical phenotypes and outcomes in juvenile dermatomyositis (JDM) have been defined by various myositis-specific autoantibodies (MSAs) [1, 2]. One of the recently described MSAs associated with dermatomyositis (DM) is targeted against small ubiquitin-like modifier 1 (SUMO-1) activating enzyme (SAE) [3]. Anti-SAE autoantibody-positive myositis patients have been reported in Caucasian (6–8%) and Asian adults (1–3%) [3–8] and are associated with severe cutaneous disease, progressive muscle disease with dysphagia, fever, and weight loss [9, 10]. It has also been reported that anti-SAE autoantibody-positive patients were at a lower frequency in the juvenile population (< 1%) [11, 12]. It is rare in children, with the details of its clinical course often unknown. We report an anti-SAE autoantibody-positive JDM patient with interstitial lung disease (ILD).

## Case presentation

**Fig. 1:**
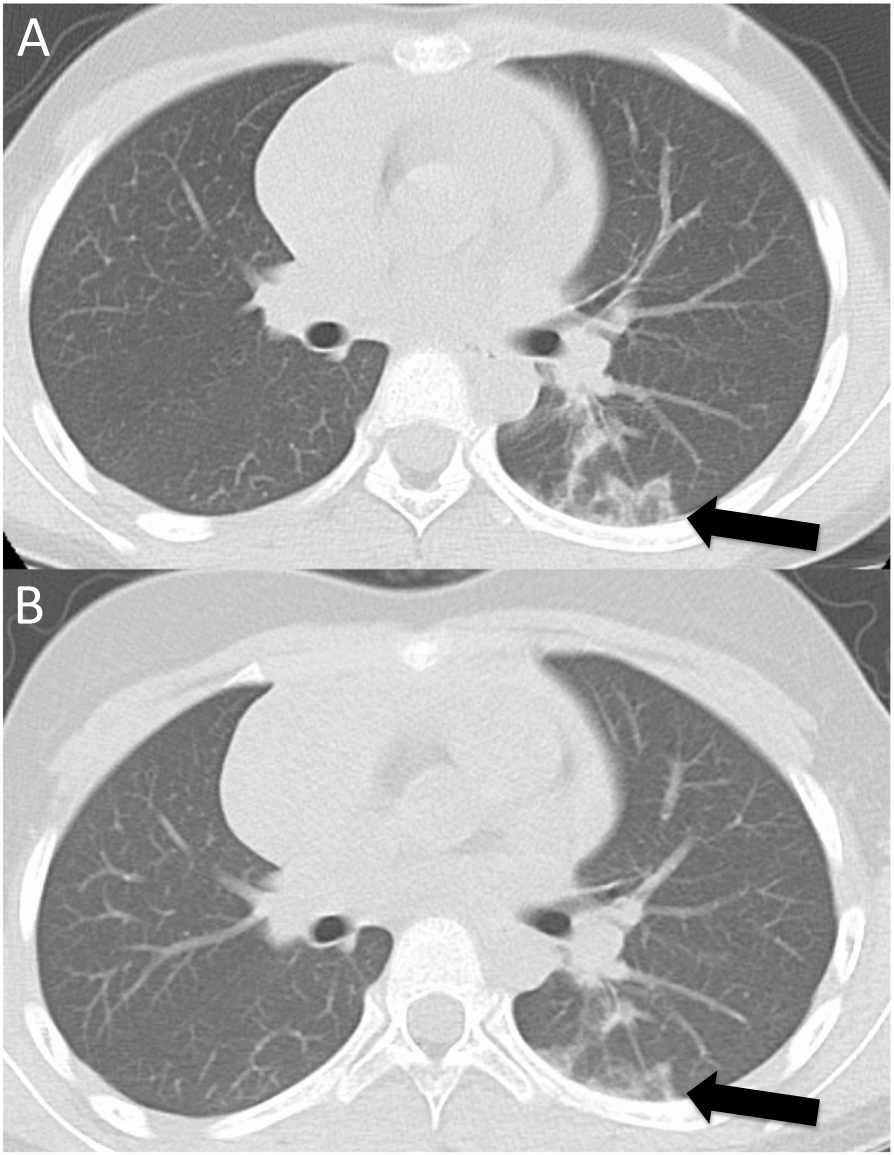
Chest computed tomography (CT) findings. **a** A ground-glass opacity was observed in the left segment 6 under the visceral pleura before treatment. The arrow shows the ground-glass opacity. **b** Ground-glass opacity was slightly improved but not progressive 18 months after treatment initiation. The arrow shows the ground-glass opacity

**Fig. 2:**
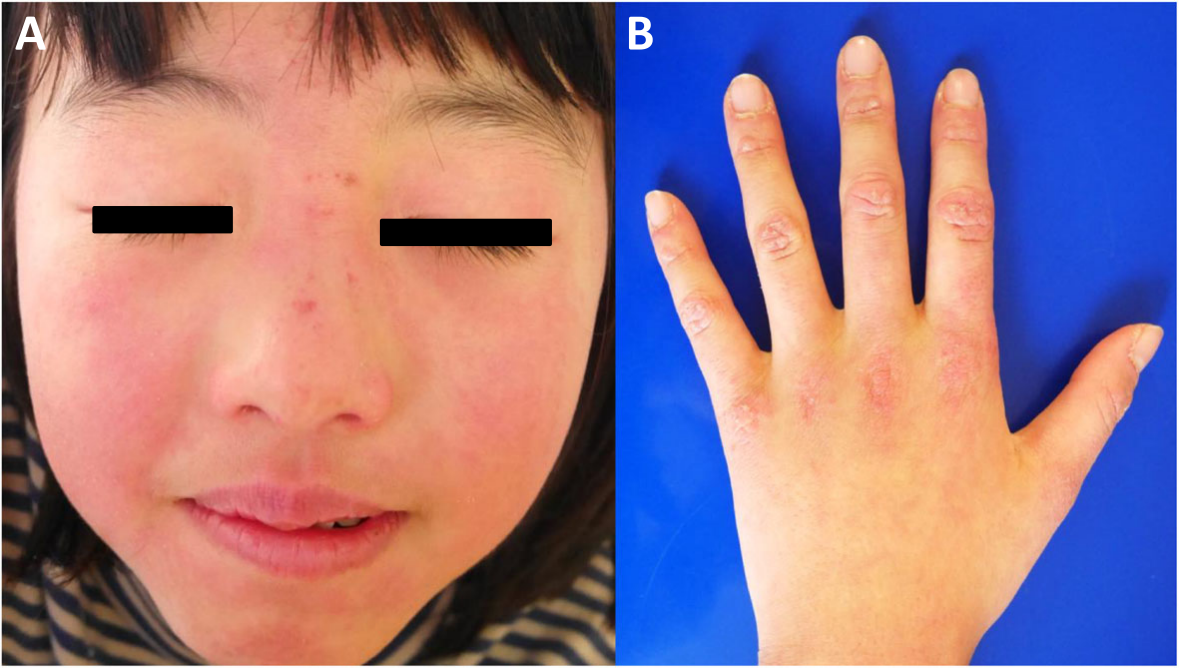
**a** Facial erythema and heliotrope rash. **b** Erythematous papules over the dorsal side of the interphalangeal joints of left hand

**Fig. 3:**
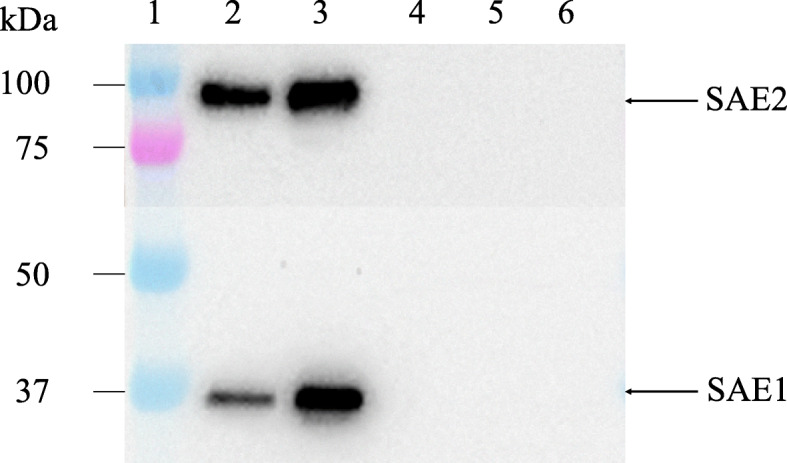
Immunoprecipitation-Western blotting of anti-SAE autoantibody. Lane1: molecular weight marker, lane2: serum from the patient, lane3: positive control (serum with anti-small ubiquitin-like modifier 1 activating enzyme (SAE) autoantibody), lane4–6: negative controls (anti-transcriptional intermediary factor-1 (TIF1) gamma autoantibody; Lane4, anti-melanoma differentiation-associated gene 5 (MDA5) autoantibody; Lane5, anti-nuclear matrix protein 2(NXP2) autoantibody; Lane6)

The patient, an 8-year-8-month-old Japanese girl, first manifested bilateral eyelid edema at 8 years 0 months and facial erythema 2 months later. At 8 years 4 months, she had dry cough and papules with erythema on the dorsal side of the interphalangeal joints of both hands. Her facial erythema gradually worsened and did not improve with topical steroids. She was referred to our department at 8 years and 8 months because of positive antinuclear antibodies and a chest computed tomography (CT) scan showing ground-glass opacity under the visceral pleura (Fig. 1a). At the first visit to our department, she had a typical heliotrope rash and Gottron’s papules (Fig. 2), with no fever or weight loss. Her childhood myositis assessment scale (CMAS) score was normal (51/52) and there was no clinical evidence of myositis, muscle weakness, or myalgia. Magnetic resonance imaging (MRI), including T2 weighted image (T2WI) and short TI inversion recovery (STIR), of the lower extremities showed normal findings without any muscle edema or myositis. An electrocardiogram and an echocardiogram showed no abnormalities. She had mild dry cough, without signs of respiratory distress, including decreased oxygen saturation. Laboratory tests showed no elevation of inflammatory markers (Table 1). Although her serum creatine kinase (CK) level was normal (91 IU/L, upper limit of normal < 163), her aldolase level was slightly elevated (9.9 U/L, upper limit of normal < 6.1). Serum Krebs von den Lungen-6 (KL-6) level was mildly elevated, serum ferritin level was normal. Anti-P155/140 (transcriptional intermediary factor-1: TIF1), anti-melanoma differentiation-associated gene 5 (MDA5), anti-Mi-2, and anti-aminoacyl-tRNA synthetases (ARS) including anti-Jo1autoantibodies were all negative in the enzyme-linked immunosorbent assays commercially available in Japan. And also anti-nuclear matrix protein 2 (NXP2) (MJ) autoantibody was negative in the immunoprecipitation- Western Blotting. Serum anti-SAE autoantibodies were detected using immunoprecipitation—western blotting (Fig. 3) [4, 13]. The details of the methods are shown in Supplemental File.

**Table 1:** Laboratory findings on admission

WBC	4800	/μl	AST	27	U/l	C3	100	mg/dl
Neutrophil	33	%	ALT	20	U/l	C4	19	mg/dl
Lymphocyte	58	%	LDH	296	U/l	CH50	45	U/ml
Hb	13.1	g/dl	CK	91	U/l	ANA	1:80	
PLTs	23.3	/μl	Aldorase	9.9	IU/L			
ESR	7	mm/hr	CRP	0.1	mg/dl	anti-SAE Ab	positive	(IP-WB)
			KL-6	646	U/ml	anti-NXP2 Ab	negative	(IP-WB)
PT (INR)	0.93		ferritin	33	mg/ml	anti-MDA5 Ab	negative	(NR < 32 titer index, EIA)
APTT	32.8	sec	IgG	1287	mg/dl	anti-ARS Ab	negative	(NR < 25, titer index, EIA)
FDP	1.0	μg/ml	IgA	133	mg/dl	anti-TIF1 Ab	negative	(NR < 32, titer index, EIA)
D-dimer	0.9	μg/ml	IgM	87	mg/dl	anti-Mi-2 Ab	negative	(NR < 53 titer index, EIA)

*Abbreviation: Ab* autoantibodies, *ALT* alanine aminotransferase, *ANA* antinuclear antibody, *APTT* activated partial thromboplastin time, *ARS* aminoacyl tRNA synthetase, *AST* aspartate aminotransferase, *CH50* complement hemolytic activity, *CK* creatine kinase, *CRP* C-reactive protein, *C3* complement component 3, *C4* complement component 4, *EIA* enzyme immunoassay, *ESR* erythrocyte sedimentation rate, *FDP* fibrin degradation products, *Hb* hemoglobin, *IgA* immunoglobrin A, *IgG* immunoglobrin G, *IgM* immunoglobrin M, *IP-WB* immunoprecipitation Western blotting, *KL-6* Krebs von den Lungen-6, *LDH* lactate dehydrogenase, *MDA5* melanoma differentiation-associated gene 5, *NR* normal range, *NXP2* nuclear matrix protein 2, *PLTs* platelets, *PT (INR)* prothrombin time (international normalized ratio), *SAE* small ubiquitin-like modifier activating enzyme, *TIF1* transcriptional intermediary factor 1, *WBC* white blood count

The patient met the 2017 American college of rheumatology/European league against rheumatism classification criteria for adult and juvenile idiopathic inflammatory myopathies [14] and was diagnosed with juvenile amyopathic DM complicated by ILD. She received two courses of methylprednisolone pulse therapy followed by oral corticosteroid (20 mg/day, 0.7 mg/kg/day) and cyclosporin A (150 mg/day, 5 mg/kg/day) administration. We gradually reduced the corticosteroid dose as her skin rash improved after treatment initiation. There were no new muscle symptoms, dysphagia, calcinosis, arthralgia, joint contractors, lipodystrophy, lipoatrophy, or periungual capillary changes during the follow-up period. There was no exacerbation of abnormal findings on chest CT scan (Fig. 1b) at 18 months from initial treatment, and no increase in serum KL-6 levels during follow-up. There was no flare of disease by the age of 10 years and 7 months, which was 24 months after treatment initiation. The patient continues to be prescribed oral corticosteroid (4 mg/day, 0.1 mg/kg/day) and cyclosporin A (50 mg/day, 1.25 mg/kg/day) at 24 months after treatment initiation.

## Discussion and conclusions

Most JDM patients have MSAs alone or those associated with specific clinical phenotypes [1, 2]. Although several phenotypic features are similar in both adult and juvenile MSA patients, some important differences exist [15]. For example, the association of an increased risk of malignancy with anti-p155/140 (TIF1) autoantibody is significant in the adults, whereas the association has not been shown in children [1, 16]. The frequency of each MSA differs between adult DM and JDM patients. Anti-synthetase autoantibodies are positive in 20–30% of adults and are less than 5% in juveniles [2, 15]. On the other hand, anti-NXP2 (MJ) autoantibody is rare in adult patients, while it is positive in 20–25% of juveniles [2], which is the second most frequent MSA in JDM. There are also differences in the rate of MSAs positivity between races. Anti-MDA5 autoantibodies are more common in the Asian population, and anti-SRP autoantibodies are seen primarily in the African American teenage girls [17, 18]. Although the same MSAs are present, the prevalence can vary by age and race, and identifying the clinical characteristics of children with MSAs is crucial to estimate prognosis and other aspects of the disease.

Anti-SAE autoantibody is the recently identified MSA first reported in 2007 by Betteridge et al. [19]. The target autoantigens are the SUMO-1 activating enzyme heterodimer consisting of 40 kDa SAE1 and 90 kDa SAE2 [3, 19]. The frequency of anti-SAE autoantibodies positivity in adult DM patients has been reported to be 5.5–8% in the European cohorts and 1.5–3.0% in the Asians [3–8]. The clinical presentation at the onset of the illness was characterized by severe cutaneous symptoms and mild myopathic symptoms. During follow-up, patients showed the progression of myositis, dysphagia, and systemic symptoms, such as fever, weight loss, and increased inflammatory markers [3, 4, 7, 13]. In addition, a study on Asian adults showed complications of interstitial pneumonia [4, 13, 20, 21]. The frequency of ILD in the Asian adult cohort was higher than that in the Western cohort [22]. And their ILD was reported not a rapidly progressive type [4, 13, 20].

Few pediatric cases with anti-SAE antibody have been reported to date, with less than 1% of the juvenile myositis cohort. Only 3 patients (0.7%) had anti-SAE autoantibodies among 379 juvenile myositis patients in the United Kingdom (UK) cohort [11]. Of the three patients, two presented with typical rash and limited or no muscle involvement but subsequently developed weakness and raised muscle enzymes. Skin diseases were persistent in both patients. In contrast, the third patient presented with seven-month history of myalgia and weakness with no rash. Myositis was diagnosed based on elevated muscle enzymes with consistent MRI and muscle biopsy findings. This patient developed typical cutaneous features of JDM 2 years later [11].

Our patient showed typical cutaneous findings of JDM, no muscle weakness, and ILD. Although typical cutaneous symptoms have been previously reported in juvenile patients with anti-SAE autoantibodies, no juvenile patients with ILD have been reported. Our patient had non-rapidly progressive (RP) type ILD, similar to the adult Japanese patients with this autoantibody [4, 13, 20]. We have shown that the Asian patients with anti-SAE autoantibodies could have ILD, even in juvenile patients. There have been few reports of pediatric patients from Asia. An association between anti-SAE autoantibody and HLA-DRB1*04-DQA1*03-DQB1*03 haplotype in adult patients with DM has been reported [3], possibly as one of the reasons for the racial differences in phenotype and frequency of these MSA-positive patients. Although the patient had no dysphagia during the 24-month follow-up, other symptoms were similar to those reported in adults, and therefore, we must be cautious about whether muscle weakness or dysphagia will occur in this patient in the near future.

In the Japanese patients, the frequency of anti-MDA5 autoantibody-positive patients is even higher in JDM patients [23]. Adult DM or JDM with anti-MDA5 autoantibody-positive patients are often complicated with ILD, which usually shows an RP type with poor prognosis [18, 23]. Therefore, combination therapy using three immunosuppressive agents, including corticosteroids, calcineurin inhibitors, and cyclophosphamide, is recommended from the early stage of illness for adult DM patients with ILD [24]. When a patient is complicated with ILD, we tend to opt for potent immunosuppressive therapy to improve the prognosis. However, depending on the differences in MSAs, the ILD may not show the RP type, as observed in this patient with anti-SAE autoantibody. Prognostic factors for RP-ILD include positive anti-MDA5 autoantibody, the deterioration of CT findings on a weekly basis, and elevated serum ferritin, KL-6, and IL-18 levels [18, 25]. For patients complicated with ILD without these prognostic factors, the possibility of non-RP-ILD should be considered along with the possibility that combination therapy of immunosuppressive agents may be over-treating these patients. As in our patient, JDM patients with ILD should be carefully evaluated before making appropriate treatment choices.

The precise role in disease pathogenesis or the difference in the frequency between adults and children of this autoantibody are not well understood. However, usually, patients with a specific MSAs are relatively homogeneous in clinical manifestations, response to therapy, and prognosis. This case report might help the better understandings of the clinical course or prognosis of this anti-SAE autoantibody positive patients and further studies are required to understand well in this autoantibody.”

We report a patient with anti-SAE autoantibody-positive JDM complicated by ILD. The patient had no progression of muscle symptoms and dysphagia during a 24-month follow-up period, which differs from the previous reports in adult patients with MSA. There have been no previous reports of pediatric patients complicated with ILD. However, ILD identified in our patient was not a RP type and did not require a cytotoxic agent. Further studies are needed to determine the characteristics of the clinical course in pediatric patients with anti-SAE autoantibodies.

## Supplementary Information

**Additional file 1.**

## Data Availability

Not applicable.

## Abbreviations

ACR: American college of rheumatology
ANA: Antinuclear antibodies
CK: Creatine kinase
CMAS: Childhood myositis assessment scale
CT: Computed tomography
DM: Dermatomyositis
EULAR: European league against rheumatism
HLA: Human leukocyte antigen
IL: Interleukin
ILD: Interstitial lung disease
JDM: Juvenile dermatomyositis
KL-6: Krebs von den Lungen-6
MDA5: Melanoma differentiation-associated gene 5
MRI: Magnetic resonance imaging
MSAs: Myositis-specific autoantibodies
NXP2: Nuclear matrix protein 2
RP: Rapidly progressive
SAE: Small ubiquitin-like modifier 1 activating enzyme
SRP: Signal recognition particle
SUMO-1: Small ubiquitin-like modifier 1
TIF1: Transcriptional intermediary factor 1
UK: United Kingdom
