# Supplementary material for: Anti-SAE autoantibody-positive Japanese patient with juvenile dermatomyositis complicated with interstitial lung disease - a case report
Source: Pediatr Rheumatol Online J. 2021 Mar 19;19:34. doi: 10.1186/s12969-021-00532-2 (PMC7980636; doi:10.1186/s12969-021-00532-2)
Supplement: Supplementary file 1 — Additional file 1. [file 12969_2021_532_MOESM1_ESM.docx]

Supplemental information

Methods:

Twenty microliters of the sera were combined with 4 mg of protein A-Sepharose beads (GE Healthcare) in IPP buffer (10mM Tris-HCl, pH 8.0, 50mM NaCl, 0.1%4-Nonylphenyl-polyethylene glycol [BioVision]) for 2 hours. Antibody-bound Sepharose beads were washed with IPP buffer, and incubated with extracts of 1 x107 K562 cells (ATCC) at 4 ℃ for 2 hours. The precipitated proteins were washed five times, fractionated by Sodium dodecyl sulfate-polyacrylamide gel electrophoresis (SDS-PAGE) using 10% polyacrylamide gel, and then transferred onto nitrocellulose membranes using a wet transfer apparatus (Mini Trans-Blot® Cell, BioRad). To detect SAE1 and SAE2, the membranes blocked with 5% skim milk were incubated with murine anti-human SAE1 monoclonal antibody (Abnoba) and rabbit anti-human SAE2 antibody (BETHYL), respectively, overnight at 4 ℃, and incubated with peroxidase-labeled goat anti-mouse or rabbit IgG polyclonal antibodies (Santa Cruz Biotechnology) after washing with tris-buffered saline with Tween 20. Then, they were visualized using an enhanced chemiluminescence kit (SuperSignal® West Pico, Thermo FisherScientific).
